# Qualitative study of the feasibility and acceptability of implementation, and potential mechanisms of Learning Together for Mental Health, a whole-school intervention aiming to promote mental health and wellbeing in secondary schools

**DOI:** 10.1186/s40814-024-01563-8

**Published:** 2024-11-15

**Authors:** Neisha Sundaram, Oliver Lloyd-Houldey, Semina Michalopoulou, Steven Hope, Joanna Sturgess, Elizabeth Allen, Rosa Legood, Stephen Scott, Lee D. Hudson, Dasha Nicholls, Deborah Christie, Russell M. Viner, Chris Bonell

**Affiliations:** 1 Department of Public Health, Environments and Society, London School of Hygiene & Tropical Medicine, 15-17 Tavistock Place, London, WC1H 9SH UK; 2 Population Policy and Practice Research and Teaching Department, UCL Great Ormond Street Institute of Child Health, 30 Guilford Street, London, WC1N 1EH UK; 3 Department of Medical Statistics, London School of Hygiene & Tropical Medicine, Keppel Street, London, WC1E 7HT UK; 4 Department of Health Services Research and Policy, London School of Hygiene & Tropical Medicine, 15-17 Tavistock Place, London, WC1H 9SH UK; 5 Institute of Psychiatry, Psychology and Neuroscience, Kings’s College London, London, SE5 8AF UK; 6 Division of Psychiatry, Imperial College London, 2nd Floor Commonwealth Building, Du Cane Road, London, W12 0NN UK; 7 Department of Behavioural Science and Health, University College London, Institute of Epidemiology and Health Care, Gower Street, London, WC1E 6BT UK

**Keywords:** Mental health, Whole-school intervention, Adolescent, Schools, Restorative practice

## Abstract

**Background:**

Despite high rates of adolescent mental-health problems, there are few effective whole-school interventions to address this. Whole-school interventions offer a feasible and sustainable means of promoting mental health. We previously evaluated the Learning Together (LT) intervention which was effective in preventing bullying (primary outcome), promoting mental well-being, psychological functioning, and reducing substance use (secondary outcomes). We adapted LT to develop Learning Together for Mental Health (LTMH) with a new menu of evidence-based actions to address mental health and an enhanced SEL curriculum.

**Methods:**

We undertook a feasibility study of LTMH, a whole-school intervention featuring needs assessment, student and staff participation in decision-making via action groups selecting actions from an evidence-based menu, restorative practice, and a SEL and resilience skills curriculum between 2022 and 2023. This article examines the feasibility, acceptability, and potential mechanisms of LTMH, qualitatively, drawing on interviews and focus groups with 49 students in years 8 and 10, and 20 staff across four state secondary schools in southern England.

**Results:**

The intervention was feasible and acceptable to implement. In terms of feasibility, the SEL curriculum was the most challenging to implement and was not prioritised by schools that had existing social and emotional learning lessons. Training and external facilitation were well-rated. Some schools struggled with the resourcing and workload implications of implementing actions from the evidence-based menu. Some aspects were not clear. Some staff were not aware that the various components worked together. Needs reports were not easy to understand for all. Students were generally supportive of restorative practice and SEL lessons. Data supported a potential mechanism involving increased school belonging and developing practical knowledge and skills to manage emotions and relationships. The intervention has little potential for harm.

**Conclusions:**

The intervention is ready for phase III trial with minor adaptations. A phase III trial of effectiveness is justified.

**Trial registration:**

ISRCTN15301591 https://doi.org/10.1186/ISRCTN15301591

**Supplementary Information:**

The online version contains supplementary material available at 10.1186/s40814-024-01563-8.

## Key messages regarding feasibility

*• What uncertainties existed regarding the feasibility?*

While previous trails indicate that schools may be prepared to engage in significant whole-school change to reduce bullying and anti-social behaviour, it is unclear if they would be willing to do so to promote mental health since this is less overtly a barrier to school functioning. It is also not clear that schools will be open to being guided by a menu of evidence-based options to implement actions. Finally, in the context of less capacity and more competing demands in schools following the COVID-19 pandemic, the ability of schools to engage in a whole-school intervention with complex change processes was uncertain.

• *What are the key feasibility findings?*

Qualitative data suggest that it is both feasible and acceptable to implement LTMH in secondary schools in England. Training and external facilitation were well-rated. However, some schools noted challenges with the resourcing and workload implications of implementing actions from the evidence-based menu. The needs assessment reports were not easy to understand for all and not all staff were aware of how the programme linked together. SEL curriculum delivery was the most challenging component and schools prioritised teaching it if they lacked existing coverage within PSHE.

• *What are the implications of the feasibility findings for the design of the main study?*

The intervention was well-received but should be adapted prior to a phase III RCT. These adaptations include modifying the timing of the study to begin in the summer term prior to intervention implementation; explaining the intervention to all staff and stakeholders in advance, including the menu of evidence-based actions to school leadership; modifying SEL curriculum materials to be more inclusive of ethnic diversity and making it an optional component for schools that cover similar topics in other lessons; and including information for parents and carers on use of restorative language in conversations with young people.

## Background

Mental health (MH) problems are the UK’s largest cause of disability [1], with around three-quarters starting before age 24 [2]. Among those aged 5–19, 13% have at least one MH disorder [3]. Schools influence MH in multiple ways including through exposure to prosocial or antisocial peers, bullying, social-support networks, and other aspects of school culture [4, 5]. ‘Whole-school’ interventions include environmental and curriculum components and have broad effectiveness across different outcomes [6]. A key aspect is increasing student engagement with school, particularly among the most disadvantaged [7, 8]. Yet most existing whole-school interventions have focused on behaviours such as bullying and substance use, with MH only assessed as a secondary outcome [9].

We previously evaluated the Learning Together (LT) intervention in a cluster trial across 40 English secondary schools from 2014 to 2017 [10]. LT was a multi-component whole-school intervention aiming to modify the school environment to reduce bullying and anti-social behaviour. The key elements were: a survey of students to inform needs assessment report (NAR); action groups (AG) comprising staff and students to review NARs, plan and coordinate local delivery, and rewrite school behaviour policies and rules supported by an external facilitator; training of all school staff in restorative practice (RP), which aims to identify harm and restore relationships in response to conflict; and a social and emotional learning (SEL) classroom curriculum. We found significant benefits of LT reducing bullying victimisation (primary outcome), improving mental wellbeing and health-related quality of life, and reducing psychological distress and substance use (secondary outcomes), with high cost-effectiveness [10].

Effect sizes for impacts on MH outcomes were approximately 0.1SD. These occurred despite the limited intervention focus on MH, other than through the SEL curriculum which was poorly implemented and therefore unlikely to have contributed to impact. This suggests the possibility that modifying LT to address MH more directly may increase the effects on such outcomes. This paper reports on the implementation and potential mechanisms of the Learning Together for Mental Health (LTMH) intervention. LTMH retains the key components of LT but also gives schools an improved SEL curriculum and new tools to make locally owned, needs-driven choices from a menu of evidence-based actions to promote student MH [11, 12].

While previous studies suggest that whole-school interventions are feasible and acceptable in schools and that AGs can successfully improve staff-student relations and drive school-environment changes [13, 14] it cannot be assumed that this will apply to LTMH because of the modifications described above. While schools may be prepared to engage in significant whole-school change to reduce bullying and anti-social behaviour, they may not be prepared to undertake such changes to promote MH since this is less overtly a barrier than bullying and anti-social behaviour to school functioning. It is also not clear that schools will be open to being guided by a menu of evidence-based options instead of having the freedom to implement any actions they see fit as was the case with LT. While it is possible that schools may be more open to addressing MH in a post-pandemic context of increased student needs [15, 16], schools may have less capacity and more competing demands, limiting their ability to engage in complex change processes [17].

Studies of implementation are increasingly informed by theory. The general theory of implementation (GTI) is especially useful in describing the processes by which interventions are delivered and normalised within settings [18]. It suggests that implementation is enacted through processes of sense-making (stakeholders understanding an intervention); cognitive commitment (to participation in delivery); collective action (shared delivery); and reflexive monitoring (reflection on progress). These processes may be affected by features of the intervention (its potential workability and integration), the personnel involved in implementation (individual intentions and collective commitments), and the institution (available material and cognitive resources, roles, and social norms).

Orientated towards the above areas of concern and informed by the GTI [18], this study explores processes of LTMH implementation to address the following research questions:

1. Is it feasible and acceptable to implement LTMH in secondary schools in England, and what refinements, if any, are advisable?
2. What do qualitative data suggest in terms of intervention mechanisms and refinements to programme theory and theory of change?
3. How do contextual factors appear to influence implementation, receipt, and mechanisms of action?
4. Are any potential harms suggested and how might these be reduced?

Quantitative results on intervention fidelity, reach and acceptability, including progression criteria, are reported elsewhere [19].

## Methods

We undertook a feasibility study with an integral process evaluation in four state secondary schools to test the intervention for one school year. All schools received the intervention in order to assess the feasibility of implementation across schools. The intervention targeted all students in years 7–11 (ages 11–16) in participating schools. This paper reports on qualitative data. Full details of the wider study are reported elsewhere [20].

### Intervention

Drawing on the theory of human functioning and school organisation [21], the intervention theory of change postulated that student mental wellbeing could be enhanced by improving relationships between staff and students and building student sense of school belonging, which in turn contributes to students developing ‘practical reasoning’ skills and peer affiliations supporting healthy decisions and wellbeing. The intervention involved:

i) NAR: schools are provided with needs reports based on data from the baseline student survey which assesses a wide range of mental health issues [22].
ii) AG: each school convenes, a group of approximately six staff members and six students collaborating on planning and co-ordinating intervention delivery, identifying local needs, and enabling student agency. A facilitator (from Place2Be charity) supports the school via online and face-to-face contact in convening and running the AG. Schools are encouraged to hold six meetings over the academic year. Details regarding the selection of AG members, and a structure and agenda for each meeting are provided to schools in a manual. The manual advises the appointment of a chair for the AG from the school senior leadership team and includes five more staff members including those with responsibility for PSHE, behaviour management, pastoral care, SEL delivery, and RP implementation. Diversity in age, gender, ethnicity, and educational engagement is advised in the selection of student AG members.
iii) Menu of evidence-based actions: the AG chooses activities from a menu of options evidenced to improve overall student MH/wellbeing with no/minimal cost to schools. Examples of these evidence-based actions include classroom sessions on body image and the media, dealing with exam stress activities, gender-sexuality alliances (GSA), mental health champions, peer mentoring or support programmes, and physical activity programmes.
iv) RP: restorative approaches aim to resolve conflict by facilitating those involved to identify harms, take responsibility, and improve relationships [23]. All staff receive introductory training introducing RP and in empathic and respectful communication. Some staff selected by the school received in-depth training and they are responsible for responding to major conflict and managing RP conferencing within the school. The introductory training is provided online and 2 out of 3 days of the in-depth training is provided face-to-face (L30 Relational Systems, accredited provider).
v) SEL curriculum: lessons on social and emotional resilience skills are delivered by teachers in timetabled lessons, tutor time, or whole-day sessions dependent on school timetables. Teachers receive online training to support delivery (Bounce Forward charity). The curriculum consists of six core resilience skills lessons meant to be delivered within hour-long lessons each to year 8 students.

### Recruitment, design, and methods

We aimed to recruit four state secondary schools in southern England. All were to be mixed-sex with a government school inspectorate rating of ‘requires improvement’ or higher and a non-temporary headteacher. Schools would vary by free school meal (FSM) entitlement rates (above or below average) indicating deprivation, and inspection rating (requires improvement or good versus excellent) indicating institutional capacity. We made a pragmatic decision to exclude schools with very low student numbers (fewer than 50 students per year). Recruitment occurred between January and April 2022. Schools were recruited by emails followed by phone calls with interested schools. School email addresses were obtained for all schools meeting our inclusion criteria in Greater London, Kent, Essex, Hertfordshire, West Berkshire, Oxfordshire, Surrey, Buckinghamshire, Cambridgeshire, and Bedfordshire. Attached to the email was an information sheet and commitment form which provided a detailed overview of the intervention and its possible benefits, the intervention and study timetable, expectations of schools, and a section for headteachers to sign a commitment to participate. In addition, the NIHR Clinical Research Network shared the study information sheet and commitment form with schools that met the study’s inclusion criteria included in their Northwest London Schools Research Network.

Qualitative data were collected via focus group discussion (FGD) or interviews with 3–6 staff per school (purposive selection by seniority/involvement in LTMH); and one FGD with year 8 (age 12/13) and one with year 10 (age 14/15) students per school during the summer term in June–July 2023. Student FGDs aimed to involve about 6–8 students with diversity reflecting the school profile in terms of gender, school engagement, and ethnicity. Year 8 and year 10 students were selected due to their participation in baseline and follow-up surveys, respectively, and year 8 students also received the SEL curriculum lessons. To be eligible, all students had to be sufficiently competent to consider consent for their participation. These requirements were provided to the lead contact at schools, who selected participants. FGDs and interviews were structured by guides with topics reflecting our research questions (Appendix 1) and facilitated by one female study researcher with a PhD (NS). They were audio-recorded, transcribed in full and anonymised, and stored securely on password-protected drives and files.

Qualitative data were subject to thematic analysis [24] by one researcher (NS) and reviewed by another (CB). Transcripts were coded using in-vivo inductive codes. Further coding used axial codes and constant comparison [25] to examine implementation, feasibility and acceptability, potential mechanisms of action and of harm, and how contextual factors might influence implementation and mechanisms; and refine the intervention and its theory of change. In the results section, the results are presented descriptively according to their source (staff or students) and the intervention component described. In the discussion section, we offer additional interpretations informed by the GTI [18] and realist approaches [26]. Realist approaches to evaluation aim to examine how interventions trigger mechanisms that in interaction with aspects of the local context contribute to the generation of outcomes. This is in order to move evaluation from questions merely of what works to questions of what works, how, and for whom.

Ethical approval for the study was obtained from the UCL and LSHTM ethics committees. Headteachers gave informed consent for intervention (Appendix 2). Informed written opt-in consent was sought from all research participants (Appendix 3), and for students their parents, were sent information sheets in advanced of data collection, allowing them to opt out themselves/their children. Just before data collection, participants received another information sheet and oral description of the study, and had the chance to ask questions before deciding whether to consent to participate.

## Results

We emailed 745 schools between 22 April and 5 May 2022 inviting them to participate in the LTMH intervention and feasibility study. Fifteen schools responded, indicating their interest in participating in the study, a response rate of 2% (although the denominator likely included a number of schools that would not have met our inclusion criteria). Out of these, six submitted completed consent forms. We purposively selected four schools (varying by FSM entitlement and government inspection rating) for the study. However, one of these schools dropped out of the study in September 2022 (prior to intervention delivery commencing) reporting concerns over its capacity to implement the intervention. We purposively selected another school from those indicating interest and this school joined the study late in November 2022, too late to complete a baseline survey. Quantitative results on intervention fidelity, reach and acceptability are reported elsewhere [19, 20]. Data collection and participants for the qualitative research are described in Tables 1 and 2. Mixed-gender FGDs with year 8 students were conducted at all four schools. However, FGDs with year 10 students were conducted at three schools; the coordinator was unable to schedule FGDs with year 10 students at school 4. A timeline of intervention activities is outlined in Table 3.

**Table 1:** Overview of process evaluation data collection activities

Data collection activity	School 1	School 2	School 3	School 4^a^
FGD/interview with teachers, number of staff-members	3	5	6	6
FGD with students year 8, *n* (% target)	1 (100)	1 (100)	1 (100)	1 (100)
FGD with students year 10, *n* (% target)	1 (100)	1 (100)	1 (100)	0 (0)

FGD Focus group discussion

^a^This was a replacement school, replacing a school that withdrew from the study after the baseline survey and did not participate in any training or intervention implementation. In other papers from this study, schools 1–4 are labelled as 1 and 3–5 reflecting the dropout of the original school 2

**Table 2:** Participation in qualitative interviews and FGDs

Participants	Activity	Number of participants				
		*School 1*	*School 2*	*School 3*	*School 4*	*Total*
Year 8 students	FGD	7	7	6	7	27
Year 10 students	FGD	8	7	7	0	22
Total number of students		15	14	13	7	49
School staff	Individual interview	3	0	0	1	4
School staff	FGD	0	5	6	5	16
Total number of staff		3	5	6	6	20

FGD Focus group discussion

**Table 3:** Timeline of intervention activities

Intervention activities	School 1	School 2	School 3	School 4
Social and emotional learning (SEL) curriculum training for teachers	September 2022 (live training, online)			Teachers viewed video-recordings in their own time
Action group meetings	October 2022–June 2023			February 2022–June 2023
Needs-assessment report distribution	November 2022			No baseline survey was conducted at this school as they joined the study late
Introductory restorative practice (RP) training for all-staff	January 2023	December 2022	January 2023	May 2023
In-depth RP training for selected staff	March 2024			
RP implementation	Began after introductory RP training			Began after in-depth training
SEL curriculum delivery by teacher(s) to year 8 students	April–June 2023	January–February 2023	January–May 2023	June–July 2023

More details about the intervention activities are reported elsewhere [19]

### School staff

#### Start-up and training

It was challenging to start the project at the beginning of the school year in autumn. Beginning the project in the previous spring term, so that meetings and staffing for the intervention can be planned ahead was preferred. Not many teachers, other than school leads, were aware of all the different intervention components. At one school, teachers suggested that school management needed to be clearer from the outset:

“It’s only the select people that have been involved in this programme actually that probably know about it. It’s not like a whole-school thing. I wasn’t aware… that all of this linked together… I think if more people knew then it would have a stronger force behind it.” (FGD, school 3)

The RP training was generally well-rated by staff. All participants described feeling confident to begin teaching and practicing RP after the in-depth training. The first two of the three training days being in-person was considered important for learning and building rapport. The only critical feedback received was a request for more time practising skills. The introductory RP training received more mixed feedback. While most staff reported finding the training useful, the lead at school 1 thought it was not delivered in the most effective manner, being held online and with insufficient practical focus:

“It wasn’t enough. There was kind of a lot of pre-waffle and then when you got to the stuff that was really important about, well actually, what does this look like in practice, how do we do it, [it stopped] and that’s what we need.” (Interview, school 1)

Some schools overcame this challenge by having those trained in-depth share information with other staff. A teacher from school 3 noted how this approach enabled their department to revise its detention policy.

The curriculum training attended by teachers was generally well-received. The main feedback received from staff in schools 1, 2, and 3, who attended the training session, was that it could have been condensed and taught over a shorter duration as it was a challenge to spare teachers from lessons; it felt awkward for some teachers to be trained alongside teachers from other schools; and more training on how practically to deliver this content would have been useful.

#### Needs-assessment reports

Schools 1, 2, and 3 received NARs and found these useful in informing AG meetings. For example, at school 2, this was helpful in developing a school-wide strategy. However, a teacher in school 1 felt that the NAR was too dense:

“When I get lots of data on a page, I kind of get a bit lost. So it would have been nicer to have had some more sort of clearer concise sort of graphs or charts with the information on there. I did find it a little bit overwhelming to look at.” (Interview, school 1)

At this school, the AG chair also wondered whether student reports reflected real need, especially when school data suggested better outcomes than the local-authority average.

#### Action groups

AGs were implemented in all schools and are popular among staff. External facilitation was considered invaluable for ensuring meetings took place. In one school, staff had unsuccessfully previously tried to convene groups of students for a similar purpose, but LTMH’s structure helped ensure meetings took place regularly. Facilitator supportiveness and direction were appreciated to ensure schools moved from discussion to action. All schools reported that student voice at AGs was essential:

“If it’s going to work, the students that go need to feel that they are being listened to and that their input matters. If they’re just attending and… doing what the teachers want…, they’re not going to buy into that.” (Interview, school 1)

Staff reported that students were keen participants at the AGs. A teacher at school 3 thought that encouraging student voices empowered them:

“It’s allowed them to feel that they’ve had an impact, which has been nice and actually there are some other students in the school that have asked if we’re doing it again next year and have said that they would like to be involved if we’re going to do something like it next year.” (FGD, school 3)

Many students attended during lunch or after school. Most staff thought AGs were diverse and allowed meaningful sharing in a safe environment:

“They work really well together as a group when it’s very, at times it was a much more representative group from across the year-groups, from a range of, you know all of the various diversities… And you know that kind of safe space for them to share their experiences.” (Interview, school 1)

AGs appeared to have the potential to modify relationships between teachers and students because each saw the other in a different context and perspective, as one teacher explained:

“They very much appreciated that they were seeing teachers in a different light and having these conversations with them and having a teacher listen to them in a different kind of way.” (FGD, school 2)

When probed about intervention mechanisms and whether these involved increased student sense of school belonging, there were mixed views. Some thought that increasing a sense of belonging among students might be an intervention mechanism. However, one teacher felt that the intervention was not advertised widely enough for it to generate widespread improvements in belonging. However, another described processes at AGs where student suggestions were implemented, which prompted other students to talk positively about these actions, potentially generating wider impacts:

“So it’s not just about the people that are involved, but it’s the message that that then sends out more widely to the wider school community.” (Interview, school 1)

Among a few staff, there was a concern that AGs did not always include those students who most needed to be heard. AGs varied in their approach to recruiting members. School 2 was working towards a wellbeing award. It found that students who had been appointed as MH ambassadors for that award slotted in easily as AG members alongside interested staff. However, this did not achieve a representation of students across all year-groups or diversity with respect to school engagement. Another school opened recruitment up to all students with students applying via an email confirming they wanted to be a part of the AG. This aimed to ensure students were committed for the whole year.

Some staff were concerned that some students selected for AGs may not be best placed to implement actions, as the AG chair at school 1 explained:

“I think the problem is that you choose people before you know what the actions are going to be and then the actions don’t necessarily suit that group of people, in terms of how to deliver it.” (Interview, school 1)

Finding a time for students and staff to meet posed a challenge for all schools. School 4, which held only three of the recommended six meetings, found this especially challenging. School 2 held their AG meetings after school, which was easier to schedule as it did not require taking students out of lessons, but it meant that younger students or those who took the bus home could not attend. School 1 had students travelling to school from afar so meetings after the school day were impossible. This school ensured meetings could be scheduled with good attendance by having them at lunchtime and providing lunch to participants.

In terms of the menu of evidence-based actions, school 1 felt that the resource implications of implementing some actions made them unfeasible.

“The list of things that you could do, are almost all school-led and/or have financial implication. So for example, the mental health first aiders is not a bad plan, but you’ve got to pay for the training.” (Interview, school 1)

The lead explained that the school did not expect the actions chosen to require the school’s own resources:

“We are forever broke. We’ve not ever, ever got any time to do any of the things we want to do… Then I guess you know looking at the kind of menu of options, when you look really closely at the menu options, they all are things that we are required to do. It’s all going to come from school, you know it’s all going to come from staff time.” (Interview, school 1)

Staff in school 3 also noted that the resultant actions placed an increased workload on AG members. They suggested delegation of implementation of actions to people outside the AG as a solution.

#### Restorative practice

Staff reported the implementation of RP to be feasible. Staff at all schools voiced support for using RP to resolve conflict. Staff in school 3 said RP was working very well at their school and some noted using it every day. There had been no push-back and all staff-members were supportive.

Staff commented on how RP could be effective in resolving conflict between students. One staff-member explained:

“I had one the other day that we’ve done a follow-up meeting following the RP conversation… Both students have said things have been better since we did that. That’s brilliant.” (Interview, school 1)

A teacher from another school commented on its benefits of healing and reintegration:

“If there’s an issue between children, they are going to have to get on with each other at some point. They’re going to go back into lessons with each other at some point. They’re going to see each other in the corridor at some point. So something like restorative justice allows that and to heal, hopefully, and reflect upon situations and then be reintegrated back into schools or lessons.” (FGD, school 2)

There were several themes concerning the mechanisms by which RP might achieve these impacts. It could help teachers improve their communication, as one explained:

“What I learned there has definitely improved the way I approach scenarios, even the language.” (FGD, school 2)

Teachers also said that their using respectful and empathetic language encouraged students to articulate their problems better. Teachers in school 3 said RP helped them feel more confident in dealing with conflict resolution. RP also provided a clear structure, helping newer staff to address conflicts:

“This allows us to have something more of a specific structure that particularly new members of staff can use and utilise. Rather than just kind of pick up as they go along.” (FGD, school 2)

Other possible intervention mechanisms noted were improved inclusivity and interaction between students, and students learning to speak more thoughtfully, which may reduce abusive banter, marginalisation, and bullying.

Some schools were keen to introduce elements of RP to parents to use with their children. One teacher described sharing information with individual parents:

“Sometimes the parents are, they are struggling with their children and I would even then [say]… ‘Okay, how about you try this method, have we tried the circle method, have we tried to do this, how about the language you use?” (FGD, school 2)

All schools decided to involve students in some way within RP. School 4 was considering training sixth formers to help resolve low-level conflict for year 7 s and 8 s. School 1 had decided that it would be most useful for RP to be student-led. This was in order to improve peer interactions and ease staff workload. However, some staff in school 1 felt students were sometimes critical of restorative approaches and needed to be reassured about their value:

“Students think harsher sanctions should be put in place all the time… In short, yes they want it to be more punitive... What they don’t want to feel is that someone has got away with it…We need to show students why it’s effective and how it’s effective, I think before we then roll it out.” (Interview, school 1)

Teachers across schools said that ideally, they would have initiated RP at the start of the academic year rather than mid-year (as required in this study). Schools were careful not to rush the roll-out of RP, to ensure its sustainability. A senior-leadership-team (SLT) member at school 1 explained that, while restorative approaches had been adopted by many staff and RP processes had been implemented by the pastoral team, it was not yet being universally used because they needed to be properly integrated:

“What we’ve tried to avoid doing is implementing something that every member of staff has to use until… we are crystal clear on what language we want to use there, how do we want them to use it and how do sanctions form part of that as well. Because we’ve made quite a few changes to our behaviour system and we need to embed that.” (Interview, school 1)

Commenting on overall feasibility, one school noted that prioritising RP implementation against other school priorities, such as academic attainment and safeguarding, was a challenge. This school decided they did not yet have the capacity to fully implement RP:

“Our Ofsted report says you need to do x, y, z. So we have to x, y, z and adding in A is really difficult, because we’re already doing x, y, z. So what we are trying to do is a kind of more student leadership style for kind of RP mentors and then see how we can then implement it as part of our behaviour strategy moving forward.” (Interview, school 1)

Lack of time in school timetables to hold restorative meetings was noted as a challenge in all schools:

“The time that it takes to do a full restorative can be a long time. I feel that sometimes we don’t have 20 to 25 minutes to sit down with two students, when deep down I know that that should be the case, that’s what we should be doing, but physically we can’t do it. So it’s sort of having to condense the restorative into ‘Right, can we do this in five minutes, can we try and use it to settle the children down enough, so that they can go into the next lesson?” (FGD, school 3)

#### Curriculum

There were mixed views on the SEL curriculum. Teachers from schools 2 and 3, which delivered all six lessons (school 3 delivered an additional lesson too, seven in total), had positive feedback. While the content of the lessons was considered good and built on other personal, social, health, and economic education (PSHE) lessons in all schools, teachers at schools 1 and 4 thought the content provided was not sufficient for a 50-min lesson. Teachers at these schools also thought the lessons were too childish in style for year 8 students. Across schools 1, 2, and 4, staff suggested that teaching the SEL curriculum to year 7 students, would have been beneficial in laying a foundation:

“I think it’s all part of that foundation approach to their learning is let’s actually look at our character, how we are looking at our emotions and our development. And if we can start them to think about that in year 7 and then build on that year after year, then actually I think that’s a greater benefit.” (Interview, school 1)

When asked what students thought of the lessons, teachers at schools 2 and 3 thought they really benefited from them. A teacher described seeing students apply what they had learned:

“What was really nice was that a couple of the students started applying the A, B, C kind of pattern especially, to their own thoughts. So one student got kicked out of a French lesson, because she had her phone out. Then we had a conversation about it and applied A, B, C towards it and that worked, and she was able to kind of process that. Her behaviour has got quite a bit better.” (FGD, school 3)

Teachers who liked the SEL curriculum described how they considered it benefited students. The lessons were said to provide a common language for communication and enable open conversations, and reduce stigma associated with mental-health discussions:

“From when the sessions were delivering was the fact that the students are actually able to openly talk about it and not feel embarrassed or not shy about it. And just quite openly talk about it because they know the terminology.” (FGD, school 2)

The lessons were also said to help students feel less isolated in their experiences, and to have the potential to prevent larger issues from developing in the future:

“Something could be dealt with at a lower level and therefore potentially stop something growing to something bigger.”(FGD, school 2)

Staff also described how practical tips on calming down and conflict resolution could help students navigate tricky situations. Staff suggested that the curriculum be provided regularly through school and not as a one-off, so students refresh and build on their learning every year. Some staff suggested making content available to parents so they are also able to support their children.

Finding an available timetable slot in which to teach the SEL curriculum was challenging, and schools adopted different approaches. Schools 2 and 3, which successfully delivered the curriculum, did not exclusively use PSHE lessons. School 2, where all lessons were delivered to all classes, noted that their PSHE lesson already had a full agenda and was only 25 min long. They used an existing subject lesson timetable slot for the SEL curriculum but this was described as not sustainable in the long-term outside the LTMH study. School 3 used a double-lesson (a combination of subject lesson, tutor time, and PSHE slots) every two weeks for one term. The uninterrupted time to deliver the lesson with regular frequency was appreciated by teachers.

School 1 delivered only two lessons to six of their eight year 8 classes. This school decided to only use the PSHE timetable slot as they worried about potentially overburdening form-tutors with delivering the SEL curriculum. However, the SEL curriculum could only be accommodated once the essential PSHE curriculum had been covered, leading to delays. School 4 delivered three of six lessons.

Factors that facilitated the implementation of the curriculum included discussions in advance with teachers on how the lessons would be delivered and giving teachers the required materials for the lessons. Where schools did not provide the necessary materials to teachers delivering the lessons, this undermined engagement. A teacher described their experience with one lesson activity, where students were meant to build towers with marshmallows and spaghetti:

“We were only given paper. So I think financially, maybe, I don’t know if they have budget to buy marshmallows. I thought it would be interesting if they use marshmallow and has a prize in the end, it would be more engaging.” (FGD, school 4)

Another facilitator was teachers being able to facilitate open discussions with students to keep them engaged in the lessons. A good rapport was important, as an English teacher explained:

“I think maybe for a couple of teachers who don’t teach more discursive subjects, they found it a bit trickier to get that relationship going with their students. But for me it was fine, very chatty, a great class.” (FGD, school 3)

When asked about potential harms, some teachers noted that discussion of emotions in lessons might be triggering for students with unresolved past episodes or feelings. One teacher suggested that providing students with information on whom they could speak with if they felt triggered would mitigate this risk. Two staff from school 1 said that, although they did not experience any direct harms from LTMH, they worried that excessive discussion of MH might cause de-sensitisation:

“My only caveat there would be what lots of our students are telling us is, ‘I just don’t want to talk about it anymore, because this is relentless’ kind of thing. So that’s the harm that I could see that some students get so switched off by it.” (Interview, staff, school 1)

#### Broader influences on implementation

Across all schools, it was noted that implementation of LTMH was influenced by school culture and priorities:

“*I think it depends on the ethos of the school, doesn’t it? You’ve got to have that cultural approach to mental health and wellbeing and not just a ‘Oh it’s on the agenda, we need to tick the box’. I think if you’ve got a school that believes in it and sees it as being a fundamental part of the school community, then it works.” (Interview, school 1)*

SLTs prioritising mental wellbeing and the LTMH programme was considered essential for feasibility. The importance of school capacity and a lead person with the authority to effect change and the motivation to lead such a programme was also considered key:

“You’ve got to have a school that has capacity to implement things and for someone to be identified as the person driving it forwards.” (Interview, school 1)

Pressure from national policy to be academically focused was perceived to be a barrier to implementation:

“I think, the main problem with schools is what we’ve lost is when they brought in league tables and it was all about results, that’s how schools were judged… It was all about their exam results and I think that’s had a massive impact, because it’s made staff, completely focused on results. It’s all about the academic. The PSHE side of it, it's all been pushed to one side. It’s also made staff competitive for the fact that your results have got to be better than somebody else’s results.” (Interview, school 1)

Financial, human resource, regulatory, and time pressures faced by schools affected their ability to implement LTMH. The post-pandemic environment was described by several staff as one featuring greater mental-health needs among students but with inadequate resources to address these. School 1 was not able to implement all LTMH components as well as hoped due to human resource and time pressures. So they focused on the components that were of greatest interest to them, involving students in the AG, but not prioritising the curriculum. It was also noted that, while some teachers were open to LTMH, others may not be and this was a reality to be acknowledged:

“I think with any school you hit a brick wall when you’ve got staff who have been teaching for X number of years and are used to a certain way of dealing with behaviour, dealing with students and kind of feel like their job is just to teach within the hour that they have the student and they don’t need to do any of the pastoral side of it. Whereas other staff I think who are more emotionally attuned to the needs of the kids and don’t just see their job within the classroom, I think they would be more receptive to being able to implement the stuff that this programme included. And also seeing the benefit of it, not just for the student, but for them as well, in terms of achieving something within their teaching.” (Interview, school 4)

Teacher MH and wellbeing were considered paramount to successfully implementing LTMH. This was because teachers set an example to their students and teacher wellbeing could also impact students’ wellbeing, explained as follows:

“It’s a vicious cycle in a funny way, because then the teachers are obviously not in a good place and that impacts the students. So I think that’s what we’re doing as a school trying to have a big push to make sure staff are happy.” (FGD, school 4)

Protecting staff wellbeing was a concern. An SLT member at one school noted the risk of asking too much of teachers with a programme such as this one:

“Teachers, not just at our school, but nationally are saying ‘We are not counsellors, we are not mental health professionals. And we want to teach Maths, we want to teach English. And we’re happy to pick up pastorally with the kids and check in that they’re okay…’ They didn’t come into the profession to talk to young people about self-harm or about eating disorders.” (Interview, school 1)

### Students

Only one of the seven FGDs included students who had participated in AGs. Students in all other FGDs said they were not aware of the overall LTMH intervention but some of them were aware of the RP and curriculum components. One student who had participated in an AG described being actively involved and contributing to actions:

“Everyone was equal in that everyone could have their say, everyone counts.” (FGD-Y8, school 4)

At an FGD with year-8 students from school 3, although no FGD participants were AG members, some students were aware of and appreciative of the wellbeing club, which was an action that the AG had implemented.

Student accounts confirmed that most schools were implementing some elements of RP. At school 1, most students appreciated the restorative approach:

“Yeah I think so, because by the end of it they were sort of friends-ish again.” (FGD-Y8, school 1)

However, some disagreed with the approach and felt stricter measures were needed:

“Say it was a situation that you were really upset about. Then sometimes they say ‘Well, if they do it again then we can sort it out’. And it’s like sometimes they’ve already done it and you would prefer them just to try and sort it out then, so it doesn’t happen again.” (FGD-Y8, school 1)

All students in our year-8 FGDs reported finding the SEL curriculum useful. Students thought it could promote emotional literacy and empathy:

“I think it also builds empathy for other people. Because you have to get used to get thinking about other people’s perspectives. So say from like a scenario where your friend saw your message, but she didn’t respond, we can’t just go and assume that they’re trying to be rude to you. You also have to look from their perspective, that maybe the person is busy.” (FGD-Y8, school 2)

The interactive elements in the SEL curriculum were considered especially useful and engaging. Across all schools, students felt that lessons which included activities and discussion were greatly preferred to those where only the teacher talked. At school 2, the fact that the lessons also included a two-way dialogue was also appreciated:

“They were also quite interactive, because we got asked what we would do and our opinions.” (FGD-Y8, school 2)

A number of students also described finding it useful to be taught specific techniques and tools that they could then apply themselves. At school 2, students described the SEL curriculum as more applied compared to their regular PSHE lessons, which provided a broad overview but no specific techniques.

The main criticism of the SEL curriculum was that of relevance. The content and examples provided were perceived as too childish for secondary-school students by some FGD participants. The scenarios provided were not considered relevant enough, and there were suggestions to tailor them to specific year-groups as one student explained:

“So if you’re going to give a scenario to year-11s, year-10s, give them something about the GCSEs. With year-7s or 8s, um, friendship problems.” (FGD-Y8, school 3)

At a school with a high proportion of South Asian students, representation and cultural relevance were also noted as an area that could be improved.

The teacher delivering the curriculum lessons and the students’ relationship with that teacher was considered important. Some students noted not being as engaged when they were taught by a substitute teacher. The value of repetition was also emphasised. Some students believed that to consistently apply what they had learned, the lessons should be delivered on an ongoing basis, explained as follows:

“I’ve found like situations where I could apply it. But because I keep forgetting myself and I’m sure that if we keep learning about them, I will one time remember and be able to use them.” (FGD-Y8, school 3)

## Discussion

### Summary of key findings

In this section, we summarise and further interpret our findings, using realist approaches [26] and the GTI [18] to frame these interpretations.

#### Is it feasible and acceptable to implement LTMH in secondary schools in England, and what refinements, if any, are advisable?

Qualitative data suggest that it is both feasible and acceptable to implement LTMH in secondary schools in England. In terms of feasibility, the SEL curriculum was the most challenging to implement and not prioritised by schools that had existing social and emotional learning lessons. Training and external facilitation were well-rated. Some schools struggled with the resourcing and workload implications of implementing actions from the evidence-based menu. The intervention was initiated in the autumn term. This short lead-in time, undermined the workability of the intervention as it hindered schools’ capacity to mobilise the time and human resources needed for implementation. In a phase III trial, it would be preferable to notify schools of whether they are allocated to the intervention and hold initial meetings with intervention schools one term earlier so that schools have sufficient time to, using terminology from GTI, make sense of the intervention, allocate the necessary time and material resources needed and start to ensure that staff commit to delivery and develop the necessary cognitive resources for this via training. There were also problems among some school staff in terms of sense-making how the intervention components should work together. We suggest using introductory meetings as well as the RP training to explain the overall project, then initiating AGs after this training is completed. This will also ensure that AGs benefit from the enhanced communication strategies that the training enables.

The RP in-depth training provided staff with important cognitive resources, such as skills in communication and rapport, for implementing RP. In-person delivery of the in-depth RP training was the preference of most participants. RP in-depth training was originally intended to be delivered all in-person but this was not possible because schools struggled to find time to release relevant staff. We recommend in-person delivery where feasible to promote participants’ cognitive participation in this component. The RP all-staff training provided a useful introduction but there were problems with school staff sense-making what was the purpose of the RP introductory training, with some participants expecting that this would provide them with skills in running restorative conferences. We suggest switching the order so that the in-depth training is delivered first to generate interest and awareness among staff prior to the all-staff training. We also suggest providing the all-staff training to one school at a time to maximise impact.

AGs were a highly workable intervention component and provided a good structure and process for schools to achieve collective action. The external facilitation increased intervention workability by supporting schools to keep on track and focused on action. The workability of the AG to achieve collective action could be enhanced by improving guidance to ensure diversity of members including students with MH needs. The NAR aimed to promote sense-making in terms of AGs developing an appreciation of student needs but this was sometimes undermined by members’ difficulty in interpreting the reports. We recommend making NARs easier to comprehend. There were problems in one school with sense-making for the menu of evidence-based actions in that one school did not realise it would need to fund the actions. This should be made clearer at the outset.

RP made sense to participants and was something they were happy to cognitively commit to. However, there were problems with sense-making among some students, who saw RP as not taking enough action against those doing harm. The intervention should include working with students so that they understand RP and see that it is not a ‘soft option’.

SEL curriculum delivery was the most challenging component. Although students made sense of and cognitively committed to the lessons, the curriculum was interpreted among some staff and students as being more orientated towards the needs of younger students. Among some students, it was also perceived as insufficiently diverse in terms of ethnicity. Adaptations could avoid these limitations. We also recommend making the curriculum an optional component because schools only committed to teaching it if they lacked existing coverage of MH within PSHE. Schools that did not deliver it fully reported not having been provided sufficient resources for delivery and finding it challenging to fit it into their timetables. This may have been because curriculum delivery was not prioritised at these schools.

Finally, staff suggested incorporating parental information or involvement to encourage parents to support the intervention and support similar learning at home.

#### What do qualitative data suggest in terms of intervention mechanisms and refinements to programme theory and theory of change?

As theorised, our data suggest that AGs could improve staff/student relationships and could build student sense of school belonging. However, participants disagreed as to whether the work of the AG could achieve such impacts among the wider student body. One suggestion that might ensure such broader impacts are achieved is for AGs to establish wellbeing clubs or other achievable ‘quick-wins’ to signal its existence and persuade students that the school is taking steps to promote their inclusion and wellbeing.

Staff were enthusiastic about RP’s potential to enable better staff and student communication, and provide staff with the confidence and appropriate procedures for resolving conflicts. Staff thought that RP could promote healing and reintegration to the school community among the parties to a conflict.

FGDs and interviews with staff and students suggest that the curriculum could equip students with emotional-management techniques, provide a vocabulary for emotional literacy, and increase empathy. Staff accounts also suggest that lessons could reduce the stigma and isolation associated with MH problems. Some staff felt that the lessons could promote inclusivity and decrease harmful banter.

#### How do contextual factors appear to influence implementation, receipt, and mechanisms of action?

It was clear that school management capacity and culture were a critical factor in influencing implementation at every stage. All components, including curriculum delivery, required strong school capacity and the availability of human, time, and material resources to ensure collective action to implement the intervention. For example, school 2 was working towards a well-being award during the time of the intervention, and actions carried out as a result of LTMH aligned well with this effort. This appeared to encourage school commitment to the intervention. In terms of training, schools could struggle if they had insufficient staff to provide cover for colleagues attending training.

AGs worked best when the role of chair was given to a committed senior staff-member and adequately supported by good scheduling and administration. The culture and style of leadership also influenced the degree to which AGs were truly participatory. AGs worked best when there were existing school norms supportive of prioritising student and staff MH, and in which staff already had a good sense of mental wellbeing.

For RP implementation, the availability of dedicated time for teachers to hold restorative meetings was critical. The ability of teachers to model the behaviour expected of students and the examples set by adults was also important. The success of implementing the SEL curriculum depended on time resources in the form of timetable space. For the curriculum delivery, ensuring teachers were adequately prepared in terms of cognitive resources and had access to the necessary material resources, were committed to teaching the subject matter, and had good relationships with the students were considered influential to successful lessons. Schools that did not have similar topics covered in their PSHE lessons were more likely to value the curriculum.

Wider contextual factors could undermine implementation. The national educational policy could undermine this by signalling to schools that they should normatively prioritise attainment above all else. Implementation could also be undermined by the post-pandemic context of increased student educational and MH needs but insufficient resourcing. More generally, a lack of school resources could also undermine implementation.

#### Are any potential harms suggested and how might these be reduced?

No specific incidents of harm resulting from LTMH were reported by any participant. There was little evidence that LTMH could cause harm, other than occasional suggestions that lessons might be triggering for students with previous unresolved negative experiences or emotions, or that lessons might cause desensitisation among students tired of repeated discussion of MH.

#### Limitations

Our qualitative research struggled to recruit student participants who had been involved in AGs or RP because of the students that schools chose to offer for this aspect of our research. Those students who did participate in qualitative research were supportive of the feasibility and acceptability of the curriculum component. Qualitative research with school staff suggested that students found other aspects of the intervention acceptable.

#### Implications for policy and research

Our study supports progression to a phase III trial of LTMH’s effectiveness. We recommend the following modifications to intervention and evaluation of a phase III trial. The timetable for the study should allow random allocation and intervention start-up in the summer term prior to implementation in the following school year. The intervention should be adapted in various ways. Start-up meetings and initial training should explain the overall structure and theory of change of the intervention. AGs should only be initiated after such training. Training should, when feasible, be done in person. In-depth training of selected staff should precede introductory training for other staff and materials should make clear the scope of each. AG guidance should include more advice about recruiting a diversity of students including those with MH needs. NAR should be easier to understand. The menu of evidence-based actions should make clear that schools will need to fund these actions. The intervention should include work with students to understand what RP is and that it is not a soft option. The SEL curriculum should be an optional component suitable for schools lacking adequate coverage of MH in PSHE lessons. SEL curriculum materials should be adapted for use with older students and be more inclusive of ethnic diversity. The intervention should include parental information about the MH of young people and the use of restorative language in having conversations with young people.

## Conclusion

The intervention was feasible and acceptable with the SEL curriculum being the most challenging to implement. As reported elsewhere, all progression criteria were met [20]. The curriculum should be made optional and intervention materials refined to provide clearer guidance. Data supported a potential mechanism involving promoting students’ sense of school belonging, and the practical knowledge and skills to manage emotions and relationships. The intervention has little potential for harm. The intervention is ready for phase III trial with minor adaptations.

## Supplementary Information

Supplementary Material 1.

## Data Availability

The datasets used and/or analysed during the current study are available from the corresponding author on reasonable request.

## Abbreviations

AG: Action groups
FGD: Focus group discussion
FSM: Free school meal
GTI: General theory of implementation
LT: Learning Together
LTMH: Learning Together for Mental Health
MH: Mental health
NAR: Needs-assessment report
PSHE: Personal, social, health, and economic education
RP: Restorative practice
SEL: Social and emotional learning
SLT: Senior leadership team
