# Supplementary material for: Qualitative study of the feasibility and acceptability of implementation, and potential mechanisms of Learning Together for Mental Health, a whole-school intervention aiming to promote mental health and wellbeing in secondary schools
Source: Pilot Feasibility Stud. 2024 Nov 15;10:142. doi: 10.1186/s40814-024-01563-8 (PMC11566735; doi:10.1186/s40814-024-01563-8)
Supplement: Supplementary file 1 — Supplementary Material 1. [file 40814_2024_1563_MOESM1_ESM.docx]

Appendix 1

Learning Together Mental Health: Staff Focus Group Topic Guide

What are your roles at the school?

What were your roles on LT-MH?

Which components were you involved in? AG, curriculum, RP

How was each component implemented? AG, curriculum, RP

Did staff and students understand the intervention?

How engaged were staff?

How involved were students?

Did staff and students buy in to the components (AG, curriculum, RP) How about the whole intervention?

How did you implement LTMH (staff and students divide up and coordinate the work)?

How did AGs go? Were they regular? Attended by a majority? Diverse (staff/students)? Well chaired? Who spoke and who didn’t?

How were decisions made?

How did AGs choose actions? Needs assessment? Student views? Staff views? Menu of evidence-based options? Other?

Did AGs implement actions?

Did AGs reflect on their own processes? On the impact of their actions?

Would you like them to continue?

In your opinion, did your schools implement RP?

How did it work? Who used it and who didn’t?

Is it something you would like to continue using? Will your school continue to use it?

Did your school implement the resilience skills curriculum?

How did it work?

How do you think it was received?

Was it useful?

How do you think AGs, RP and curriculum may be affected by the characteristics of schools, staff and students e.g. resources, priorities, other commitments, values, workload, time, relationships

How do you think LT-MH might improve mental health?

Do you think there may be a way that LTMH implementation at your school may improve relationships, belonging and social/emotional skills? How so, in your opinion?

Does AG have a role? What?

RP’s role and what?

Curriculum’s role and what?

What about the contributions to this mechanism of the actions chosen?

Do you think LT-MH will have more impact on mental health in some schools?

Do you think it will have more impact for some students?

Do you think it will have more impact when used by some staff?

Could it cause any harms to mental health or anything else?

How do you think LT-MH worked in practice as opposed to in theory?

Do you think it actually improved relationships, belonging or social/emotional skills?

Do you think it worked better for some schools and students than others?

Learning Together Mental Health: Student Focus Group Topic Guide

*LTMH - general*

Are you aware of actions the school has been taking to improve mental health? If so, what? What have you thought of them?

Are you aware of Learning Together Mental Health?

What actions have you noticed?

What actions have you been involved in?

*Resilience skills curriculum*

Have you been taught the Resilience curriculum?

What were the lessons like? Which timetable period was it taught in? Were they taught well? Were they interesting? Did they involve discussions?

Do you think they were useful? What do you feel you got out of it? One most important thing? Do you feel you can use the lessons in real-life situations?

Would you like for them to continue?

*Restorative practice*

Has restorative practice been introduced to you at your school? If so, how?

How would you describe restorative practice? What is the purpose of restorative practice in your opinion?

Do you think restorative practice is useful? Do you feel you can use it in real-life situations

Have you been involved in restorative practice?

If so tell me about this – why/how were you involved, what was the process like? What was the result, what impact did it have on you/others involved? was is useful?

Can you think of any times when restorative practice has worked well?

What about when it has not worked well?

Has your school started using restorative practice? Would you like it to be used more widely?

*Action group*

Were any of you involved in an action group at your school?

Did you think it was a good idea?

Did staff and students know what they were meant to do on this?

How engaged were staff?

How involved were students?

What can you tell me about how staff and students engaged with each other? For example, the way they spoke to each other and the overall atmosphere or tone of the meetings. Who spoke and who didn’t? Was everyone in the group invited to speak? Was anyone put off from speaking or contributing?

How did staff and students divide up and coordinate the work?

How did the AGs go? Were they regular and well attended? Diverse (staff/students)? Well-chaired?

How were decisions made?

How did AGs choose actions? Needs assessment? Student views? Staff views? Menu of evidence-based options? Other?

Did AGs implement actions?

Were there actions that were not implemented? If yes, why were they not implemented?

Did AGs reflect on their own processes? On the impact of their actions?

Would you like for them to continue?

Those who were not involved – would you like to be?

*Closing*

Does anyone have any final thoughts to share on the Learning Together Mental Health program, Resilience skills teaching, Restorative practice, Action groups or any other mental health activities at your school?

Before we end, I’d like to go around the room and ask everyone if you could do one thing differently for the Learning Together Mental Health program what would it be?

Thank you for your time and very valuable input. It is extremely useful for the research. If you have any mental health questions please refer to the sources of help on your information sheet.

Appendix 2

Headteacher Information sheet and commitment form

Signing up for the study

Schools opting to participate in this feasibility study will have the opportunity to take part in a project to pilot a new programme to promote mental health in secondary schools. The project is built upon strong evidence that this approach improves wellbeing of students in years 7-11.

Schools which signal their interest in participation will be informed if they have been selected to take part by end of May 2022.

Benefits for the school

Participating schools will have the opportunity to implement without charge the Learning Together-Mental Health, a programme that aims to provide schools with evidence-based tools to promote young people’s mental health.

Learning Together-Mental Health supports schools’ duty to promote young peoples’ social and emotional wellbeing.

Provision includes free staff professional development, lesson plans, learning resources, bespoke information on the prevalence of mental health problems of students in the school (vs. nationally), and access to resources to support the implementation of mental health interventions in school.

Staff receive free, quality-assured professional development to deliver the resilience-based PSHE curriculum “Resilience Skills” (training and resources provided by the charity Bounce Forward) and to implement a whole-school approach to restorative practices (training and resources provided by restorative training specialists, L30).

Practical support implementing Learning Together-Mental Health will be provided by Place2Be, a children’s mental health charity with over 25 years’ experience working with young people, families and staff in UK schools.

The study will be evaluated by University College London (UCL), the top-rated university in the UK for research strength and the London School of Hygiene and Tropical Medicine (LSHTM), Europe’s leading public health institute.

The study also offers students the chance to meet and work with leading public health scientists, some of whom have played a key role in scientific advice to government on Covid-19 and other major health issues.

Timetable

- UCL researchers undertake a survey of year 7 students’ mental health and well-being (June-July 2022)
- Bounce Forward train school staff in the Resilience Skills curriculum (September-October 2022)
- Schools implement Learning Together-Mental Health intervention with Place2Be support (September 2022-July 2023)
- L30 train school staff in restorative practice (January-March 2023)
- Schools collaborate in research activities with UCL and LSHTM support (September 2022-July 2023)
- UCL researchers undertake a survey of year 10 students’ mental health and wellbeing (June-July 2023)

Learning Together-Mental Health programme components

1) Action group comprising staff and students focuses on reviewing and revising school policies and systems to promote mental health and wellbeing, supported by an external Place2Be facilitator. The Action Group reviews local needs assessment data (see component 2 below) to set priorities for improving mental health and wellbeing in the school and to guide decisions for intervention elements from a set ‘menu’ of possible evidenced-based actions that have previously been shown to work in schools (see component 3 below).

2) Needs survey of year 7 students identifies mental health and well-being needs to help the school Action Group build school commitment to the work and set local mental health and well-being priorities.

3) Menu of actions focuses on potential evidence-based interventions within the school relating to a range of areas, including body image/self-esteem, digital health, and LGBTQ+ inclusion. Choice of actions in each school will be guided by the needs assessment, based on data obtained in the needs survey. Actions are evidence-based, are practical and free/minimal cost to implement in schools.

4) Classroom curriculum delivered by school staff, addressing social and emotional skills. The Healthy Minds curriculum provides foundational resilience skills in 55-minute lessons totalling 15-18 hours over the school year. It can be used with students in Years 7-10, and each school can decide which years and classes will be offered these lessons.

5) Restorative practice delivered by school staff, helping students to resolve conflict, take responsibility for behaviour, and engage in acts of empathy and forgiveness, supported by enhanced connection with school. A subset of teachers will have in-depth training in restorative conferencing to enable them to address more serious incidents.

Learning Together-Mental Health provides schools with the following:

- An external facilitator from the children’s mental health charity Place2Be to help implement the intervention
- A curriculum focused on social and emotional skills (Healthy Minds)
- Healthy Minds curriculum training for 2 school staff (online in two sessions over 1.5 days).
- Restorative Practice training (all teachers will receive 90 minutes online training; 3–5 staff will receive more intensive training via a blend of 2 days face-to-face training and 1 day of online)
- a manual to guide the school to carry out the intervention

Schools will be expected to collaborate in the following research activities:

- A survey of year 7 students in June-July 2022. Paper questionnaires will be completed confidentially in classrooms supervised by UCL fieldworkers.
- A survey of year 10 students in June-July 2023 under the same conditions.
- Two focus groups, one with year 8 and one with year 10 students, will take place during school year 2022-23. Each will involve about 6-8 students. Two focus groups will also take place each with four staff.
- Brief, tick box logbooks to be completed by school staff implementing internal training, Action Groups, and the curriculum.

Headteacher commitment

I have read the above information and would like to give permission for my school to participate in this study.

*Headteacher name:*

*School name*

*Signed:*

*Date:*

Appendix 3

Information Sheet -

Learning Together–Mental Health student focus group

We are researchers from a university called the London School of Hygiene and Tropical Medicine (LSHTM). We have been working with your school to test out a new mental health support programme for secondary schools called Learning Together – Mental Health. This study has been approved by the research ethics committee of the London School of Hygiene & Tropical Medicine. As part of our research, you have been selected to take part in a focus group to give your views on the programme and how well it has worked in your school. The focus group is intended to help us evaluate the programme itself and not the input of staff, students or the school. You’ve been invited to this focus group because of your experience receiving some elements of the programme and not because of your mental health experiences.

What does taking part involve?

The focus group will last about one hour and will take place at school, during the school day. If you agree to take part, you will be asked about your views and opinions on the Learning Together – Mental Health programme. You will not be asked to discuss your own experiences of mental health or any other personal matters.

What will we do with the information we collect?

We will audio record the focus group and then produce a written record of what was said. We will write reports based on what we find, which will help decide how suitable the programme is for secondary schools in England and contribute to improving mental health for young people.

Will the information I provide be kept private and confidential?

The views and opinions you express in the focus group will be kept completely private and confidential by the researchers. The recording, written record of what is said and any notes from the focus group will be stored safely and confidentially in our offices on a computer that only the research team can access. The research will be published in scientific journals and shared with policy-makers. No student or school names will be included in any of these publications. Any direct quotes we use will be anonymised.

As the focus group will involve other participants we cannot guarantee that everything that is said in the group will remain private. We ask you to be mindful of this and not to disclose anything personal about yourself or anyone else to the group.

If you should say something during the focus group that leads us to believe you or someone you know may be at risk of very serious harm we will need to tell someone else about this. If this happens, we will discuss it with you first.

Do I have to take part in this research?

It is entirely up to you if you want to take part in our research. If you decide to take part, you can stop taking part at any time and you do not have to answer any questions you do not want to. There are no financial incentives to participate. We do not anticipate that the focus group will be distressing but if you do experience distress and want to stop at any point for this or any other reason this will be possible.

What do I need to do next?

If you are happy to take part in the focus group, you will be given a consent form to sign on the day. If you are not happy to take part or if you have any questions about the research please contact Neisha Sundaram who is leading the research for LSHTM (email neisha.sundaram@lshtm.ac.uk). If you have any concerns about the project, please email the lead investigator Chris Bonell ([chris.bonell@lshtm.ac.uk](mailto:chris.bonell@lshtm.ac.uk)) or the chair of the independent study steering committee Tamsin Ford ([tjf52@medschl.cam.ac.uk](mailto:tjf52@medschl.cam.ac.uk)).

Alternatively, if you would rather speak to somebody outside the research team, you can contact [XXXX] directly, who is the programme lead at your school, to let them know you do not want to participate and/or to raise any concerns or complaints about the research.

Additional sources of information, advice and support:

For further information and advice about mental health you can visit the following websites:

- The Samaritans: Someone to talk to, available 24 hours a day for confidential, non-judgmental support. Call 116 123 or visit [www.samaritans.org](http://www.samaritans.org)
- Switchboard LGBT+ Helpline: Providing information, support and referral services for lesbians, gay men and bisexual and trans people, and anyone considering issues around their sexuality or gender identity. Call 0300 330 0630 or visit http://switchboard.lgbt/help/
- Mind: Offering advice and support for anyone experiencing a mental health problem. Call 0300 123 3393 or visit [www.mind.org.uk](http://www.mind.org.uk)

If you would like to talk to someone about an issue you or someone you know is going through you can speak directly to [XXXX], who is the safeguarding lead at your school.

Childline are also available on 0800 1111 24 hours a day, every day of the year to discuss any issue, big or small.

Many thanks for your time,

Professor Chris Bonell (London School of Hygiene and Tropical Medicine)


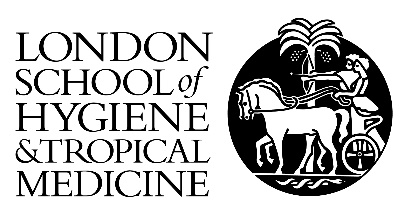


Consent form-student focus group

We are researchers from a university called the London School of Hygiene and Tropical Medicine (LSHTM). We are working on a research study to evaluate mental health support in secondary schools in England. As part of our research, you have been selected to take part in a focus group with some other students so we can find out about your experiences of mental health support in your school. You’ve been invited to this focus group because of your experience receiving some elements of the programme and not because of your mental health experiences.

What does taking part involve?

The focus group will last up to one hour and will take place at school, during the school day. If you agree to take part, you will be asked about your views and opinions on mental health support in your school. You will not be asked to discuss your own experiences of mental health or any other personal matters.

What will we do with the information we collect?

We will audio record the focus group and then produce a written record of what was said. We will write reports based on what we find. Our research will contribute to improving mental health support for young people in England.

Will the information I provide be kept private and confidential?

The views and opinions you express in the focus group will be kept completely private and confidential by the researchers. The recording, written record and any notes from the focus group will be stored safely and confidentially in our offices on a computer that only the research team can access. When we write reports about our research, we will not include names or any information that may identify anyone who takes part, or their school. Any direct quotes we use will be anonymised.

As the focus group will involve other participants, we cannot guarantee that everything that is said in the group will remain private. We ask you to be mindful of this and not to disclose anything personal about yourself or anyone else to the group.

If you should say something during the focus group that leads us to believe you or someone you know may be at risk of very serious harm we will need to tell someone else about this. If this happens, we will discuss this with you first.

Do I have to take part in this research?

It is entirely up to you if you want to take part in our research. If you decide to take part, you can stop taking part at any time and you do not have to answer any questions you do not want to. There are no financial incentives to participate. We do not anticipate that the focus group will be distressing but if you do experience distress and want to stop at any point for this or any other reason please let the researcher know.

What do I need to do next?

If you have any questions, the researcher will be happy to answer them.

If you’re happy to take part in our research, please fill in the box below.

If you would like to speak to someone about mental health or any other issue you or someone you know are going through, a list of people and organisations you can contact for information, advice and support both inside and outside school was provided on the information sheet you received recently. Another copy has been provided for you to take away today.

Full Name [please print] ……………………………………………………..…………..

I have read the attached information sheet. 🞏

I have been given the opportunity to ask questions. 🞏

I understand that I can choose to take part or not. 🞏

I understand that I can stop taking part at any time. 🞏

I agree that the focus group can be audio-recorded. 🞏

I understand that anonymised direct quotes from me may be used in

the reporting of this study. 🞏

I agree to take part in this focus group. 🞏

Signed ……………………………………………….. Date ………………………**…………**

**Information Sheet -**

**Learning Together–Mental Health staff focus group**

We are researchers from the London School of Hygiene and Tropical Medicine (LSHTM). We have been working with your school to evaluate a mental health support programme for secondary schools called Learning Together–Mental Health. This study has been approved by the research ethics committee of the London School of Hygiene & Tropical Medicine. As part of our research, we would like to invite you to take part in a focus group with other staff to discuss your experiences of the programme and how well it has worked in your school. You have been chosen to participate because of your involvement with the programme. The focus group is intended to help us assess the programme and not the performance of staff, students or the school.

**What does taking part involve?**

The focus group will take up to an hour to complete and will take place at school, during or directly after the school day. If you agree to take part, it will involve you giving your views and opinions on the Learning Together – Mental Health programme. You will **not** be asked to discuss your own experiences of mental health or any other personal matters.

**What will we do with the information we collect?**

We would like to audio record the focus group and then produce a written record of what was said. We will write reports based on what we find. Our findings will be used to assess how suitable the programme is for secondary schools in England and will contribute to improving mental health for young people.

**Will the information I provide be kept private and confidential?**

In line with ethical guidelines for research, the views and opinions you express in the focus group will be kept completely confidential by the researchers. The recording, written record and any notes from the focus group will be stored securely in our offices on a computer that only the research team can access. The research will be published in scientific journals and shared with policy-makers. No staff, student or school names will be included in any of these publications. These may contain direct quotes from participants, but these will be fully anonymised.

If you should say something during the focus group that leads us to believe you or someone you know may be at risk of very serious harm we will need to tell someone else about this. If this happens, we will always discuss it with you before we tell anyone else.

**Do I have to take part in this research?**

It is entirely up to you if you want to take part in our research. If you decide to take part, you can stop taking part at any time and you do not have to answer any questions you do not want to. There are no financial incentives to participate. We do not anticipate that the focus group will be distressing but if you do experience distress and want to stop at any point for this or any other reason this will be possible.

**What do I need to do next?**

If you are happy to take part in the focus group, you will be given a consent form to sign on the day. If you are not happy to take part or if you have any questions about the research please contact Neisha Sundaram who is leading the research for LSHTM (email neisha.sundaram@lshtm.ac.uk). If you have any concerns about the project, please email the lead investigator Chris Bonell ([chris.bonell@lshtm.ac.uk](mailto:chris.bonell@lshtm.ac.uk)) or the chair of the independent study steering committee Tamsin Ford ([tjf52@medschl.cam.ac.uk](mailto:tjf52@medschl.cam.ac.uk)).

Alternatively, if you would rather speak to somebody outside the research team, you can contact XXXX directly, who is the programme lead at your school, to let them know you do not want to participate and/or to raise any concerns or complaints about the research.

Many thanks for your time,


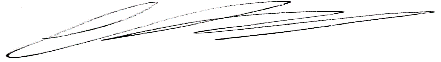


Professor Chris Bonell (London School of Hygiene and Tropical Medicine)

**
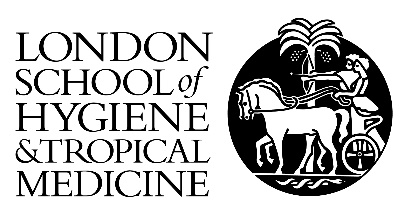
**

**Consent form for staff focus group**

We are researchers from the London School of Hygiene and Tropical Medicine (LSHTM). We are working on a research study to evaluate the Learning Together – Mental Health programme. As part of our research, we would like to invite you to take part in a focus group to find out about your experiences with this programme in your school. You have been chosen to participate based on your role in the school, not for any other reasons.

**What does taking part involve?**

The focus group will take about 1 hour to complete and will take place at school, during or directly after the school day. If you agree to take part, it will involve you giving your views and opinions about the programme. You will **not** be asked to discuss your own experiences of mental health or any other personal matters.

**What will we do with the information we collect?**

We would like to audio record the focus group and then produce a written record of what was said. We will write reports based on what we find. Our findings will contribute to improving mental health support for young people in England.

**Will the information I provide be kept private and confidential?**

In line with ethical guidelines for research, the views and opinions you express in the focus group will be kept completely confidential by the researchers. The recording, written record and any notes from the focus group will be stored securely in our offices on a computer that only the research team can access. When we write reports based on the information we collect, we will not include the names or any information that may otherwise identify anyone who takes part in the focus groups or their school. Our reports may contain direct quotes from participants, but these will be fully anonymised.

If you should say something during the focus group that leads us to believe you or someone you know may be at risk of very serious harm we will need to tell someone else about this. If this happens, we will discuss it with you before we tell anyone else.

**Do I have to take part in this research?**

It is entirely up to you if you want to take part in our research. If you decide to take part, you can stop taking part at any time and you do not have to answer any questions you do not want to. There are no financial incentives to participate. We do not anticipate that the focus group will be distressing but if you do experience distress and want to stop at any point for this or any other reason this will be possible.

**What do I need to do next?**

If you have any questions the researcher will be happy to answer them.

If you’re happy to take part, please fill in the box below.

Full Name ……………………………………………………..…………..

I have read the attached information sheet.

I have been given the opportunity to ask questions.

I understand that I can choose to take part or not.

I understand that I can stop taking part at any time.

I agree for the focus group to be audio-recorded.

I understand that anonymised direct quotes from me may be used in the reporting of this study

I agree to take part in this focus group.

Signed ……………………………………………….. Date …………………………………
